# Deciphering the role of *Enterococcus faecium* cytidine deaminase in gemcitabine resistance of gallbladder cancer

**DOI:** 10.1016/j.jbc.2024.107171

**Published:** 2024-03-15

**Authors:** Lin Jiang, Lingxiao Zhang, Yijun Shu, Yuhan Zhang, Lili Gao, Shimei Qiu, Wenhua Zhang, Wenting Dai, Shili Chen, Ying Huang, Yingbin Liu

**Affiliations:** 1 School of Health Science and Engineering, University of Shanghai for Science and Technology, Shanghai, China; 2 Department of General Surgery, Shanghai Research Center of Biliary Tract Disease, Shanghai Key Laboratory of Biliary Tract Disease Research, Xinhua Hospital Affiliated to Shanghai Jiao Tong University School of Medicine, Shanghai, China; 3 Department of Biliary-Pancreatic Surgery, Shanghai Key Laboratory for Cancer Systems Regulation and Clinical Translation, State Key Laboratory of Systems Medicine for Cancer, Renji Hospital Affiliated to Shanghai Jiao Tong University School of Medicine, Shanghai, China

**Keywords:** cytidine deaminase, crystal structure, gemcitabine, chemotherapy, gallbladder cancer

## Abstract

Gemcitabine-based chemotherapy is a cornerstone of standard care for gallbladder cancer (GBC) treatment. Still, drug resistance remains a significant challenge, influenced by factors such as tumor-associated microbiota impacting drug concentrations within tumors. *Enterococcus faecium*, a member of tumor-associated microbiota, was notably enriched in the GBC patient cluster. In this study, we investigated the biochemical characteristics, catalytic activity, and kinetics of the cytidine deaminase of *E. faecium* (EfCDA). EfCDA showed the ability to convert gemcitabine to its metabolite 2′,2′-difluorodeoxyuridine. Both EfCDA and *E. faecium* can induce gemcitabine resistance in GBC cells. Moreover, we determined the crystal structure of EfCDA, in its apo form and in complex with 2′, 2′-difluorodeoxyuridine at high resolution. Mutation of key residues abolished the catalytic activity of EfCDA and reduced the gemcitabine resistance in GBC cells. Our findings provide structural insights into the molecular basis for recognizing gemcitabine metabolite by a bacteria CDA protein and may provide potential strategies to combat cancer drug resistance and improve the efficacy of gemcitabine-based chemotherapy in GBC treatment.

Gallbladder cancer (GBC) constitutes a significant proportion of biliary tract malignancies and is recognized as one of the most lethal biliary system neoplasms (1). The 5-year survival rate associated with GBC is dismally low, varying between 5% and 15% (2). GBC usually presents no symptoms in the initial stages and is prone to aggressive metastasis, often leading to delayed diagnosis and a grim prognosis for most patients (3). Surgical resection, the only potentially curative treatment, hangs in the balance of factors such as disease stage, tumor biology, and completeness of resection (4). It is common for patients to be diagnosed with advanced diseases at presentation or to experience disease recurrence following surgery (5). Systemic chemotherapy, aimed at symptom alleviation and survival extension, is the sole available treatment for these patients (1). Current standard care for GBC features gemcitabine (2′,2′-difluorodeoxycytidine)-based chemotherapy regimens with a demonstrated survival advantage in primary treatment (6). Nevertheless, advanced GBC frequently develops resistance to traditional chemotherapeutic drugs, including gemcitabine and other agents like cisplatin, 5-fluorouracil, oxaliplatin, and capecitabine (4, 6, 7).

Cancer drug resistance is a complex process with numerous contributory factors, including the tumor-associated microbiota (8). These microbial entities residing in and around multiple cancer types boast a unique colony composition, differing substantially from that of healthy tissue (9). Their role includes manipulating the immune system, suppressing cancer-fighting immune cells, favoring tumor growth, and producing metabolites influencing cancer cells' response to chemotherapy and other treatments (10, 11, 12). Furthermore, tumor-associated bacteria can reduce drug concentrations within tumors while not necessarily impacting other organs (8).

Bacterial enzymes' influence on tumor susceptibility to chemotherapeutic drugs such as gemcitabine has been previously examined in the context of pancreatic ductal adenocarcinoma (PDAC) (8). Gemcitabine is a synthetically produced pyrimidine nucleoside prodrug. Its chemical structure features a fluorine atom replacing a hydrogen atom at the 2′ carbon of deoxycytidine, which makes it hydrophilic (13). Once transported into cells through specific nucleotide transporters, gemcitabine undergoes deoxycytidine kinase phosphorylation, resulting in gemcitabine monophosphate (dFdCMP). The following processes yield the triphosphate variant, gemcitabine triphosphate (dFdCTP), through additional enzymatic alterations. During DNA synthesis, dFdCTP can stand in for deoxycytidine triphosphate (14). Gemcitabine's incorporation into DNA bypasses the cell's standard repair system (base excision repair) (15). Such an abnormal inclusion inhibits further DNA synthesis, thereby inducing cell death (16). Gemcitabine resistance among cancer patients can stem from inherent or acquired mechanisms attributed to changes in the tumor microenvironment and the drug's metabolism (17, 18). Various cellular factors are thought to impact tumor cells' sensitivity and resistance to Gemcitabine (19). These include the nucleoside transporter hENT1, deoxycytidine kinase deoxycytidine kinase, and the 5′-nucleotidases I and III (cN-I and cN-III) (18, 20, 21, 22).

Additionally, lessening cytidine deaminase (CDA) activity, which can obstruct gemcitabine deamination, has enhanced tumor cell sensitivity to gemcitabine (23). As a zinc ion–dependent enzyme, CDA participates in the pyrimidine salvage pathway by converting cytidine and deoxycytidine into uridine and deoxyuridine. In PDAC, bacterial CDA can modify tumor susceptibility to gemcitabine by transforming the drug into its inactive metabolite, 2′,2′-difluorodeoxyuridine (dFdU), which lowers gemcitabine's bioavailability in the tumor, thereby reducing its effectiveness (8, 24).

CDA proteins are conserved among various species, from bacteria to humans (25). However, in humans, CDA primarily acts on RNA, with only a subset of enzymes acting on DNA (26). This action requires ssDNA as a substrate, not a base. Bacteria extensively exhibit two forms of CDA: a long form and a short one. The former harbors two tandem CDA domains typically structured in a dimer assembly (homodimeric CDA or D-CDA), while the latter possesses a single catalytic domain arranged in a tetramer assembly (termed homotetrameric CDA or T-CDA) (27, 28). Previously, it was reported that only bacterial D-CDA can deactivate the chemotherapeutic agent gemcitabine (8). However, in contrast, another study suggested that the short version of bacterial T-CDA can convert various N4-acylarylpyrimidines, N4-/S4-/O4-alkylarylpyrimidines, and N4-/S4-/O4-arylpyrimidine nucleosides into uracil derivatives, including capecitabine, another chemotherapy drug (29). Consequently, whether T-CDA can act on gemcitabine needs to be explored.

A previous study accentuated significant biliary microbial composition and gene function differences between GBC patients and chronic calculous cholecystitis patients. *Enterococcus faecium* was discernibly enriched and positively associated with the GBC patient cluster (30). The CDA derived from *E. faecium* (EfCDA) belongs to the T-CDA form. Whether the T-CDA from tumor-associated microbiota impacts gemcitabine resistance in gallbladder cancer remains unclear. In this study, we discovered that both EfCDA and *E. faecium* have the ability to degrade gemcitabine, which results in drug resistance in GBC cells. The crystal structure of EfCDA was determined in its apo form and in complex with gemcitabine. Mutation of key residues abolished the catalytic activity of EfCDA and restored cell sensitivity to gemcitabine. These results may help identify new targets for drug therapy and improve our understanding of the molecular mechanisms involved in CDA-related chemotherapy resistance.

## Results

### EfCDA can induce gemcitabine resistance in GBC cells

**Figure 1:**
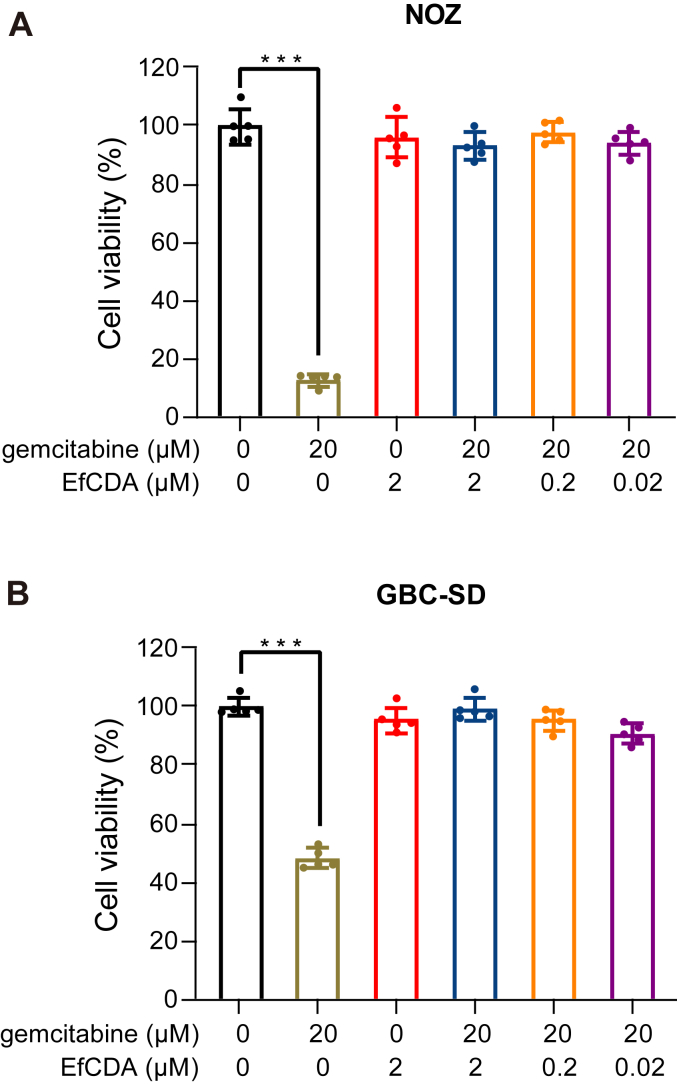
**GBC cells exhibit gemcitabine resistance in response to EfCDA.***A* and *B*, viability assays of NOZ cells (*A*) and GBC cells (*B*) were conducted using the CCK-8 assay after treatment with EfCDA and gemcitabine. NOZ and GBC-SD cells were exposed to concentrations of 0, 0.02, 0.2, and 2 μM EfCDA, 20 μM gemcitabine, or their combination for a duration of 72 h. The Student’s *t* test was employed to compare the effects of gemcitabine alone with its combination with EfCDA. Significance levels are denoted as ∗*p* < 0.05, ∗∗*p* < 0.01, and ∗∗∗*p* < 0.001. GBC, gallbladder cancer.

To determine whether EfCDA can induce gemcitabine resistance in GBC cells, gemcitabine's impact on the viability of GBC cells was initially assessed. A distinct divergence in the tolerance to gemcitabine was observed between the two GBC cell types. In comparison to GBC-SD cells, NOZ cells exhibited higher sensitivity to the drug. Specifically, when exposed to 20 μM gemcitabine, NOZ cells showed a significant 80% decrease in cell viability compared to the control group (Fig. 1*A*). In contrast, GBC-SD cells demonstrated a 60% reduction in cell viability under the same treatment conditions (Fig. 1*B*).

Interestingly, regardless of whether the medium was supplemented with gemcitabine, the pre-incubation of the purified full-length EfCDA protein in the medium for 2 h, followed by the cultivation of NOZ and GBC-SD cells in this prepared mixture, caused no significant alterations in cell growth. To further confirm the result, the viability of both cells was evaluated across three different concentrations of EfCDA proteins (ranging from 0.02 μM to 2 μM). The results consistently suggested that EfCDA may contribute to resistance to gemcitabine in GBC cells, even at a lower concentration of 0.02 μM.

### *E. faecium* can also induce gemcitabine resistance in GBC cells

**Figure 2:**
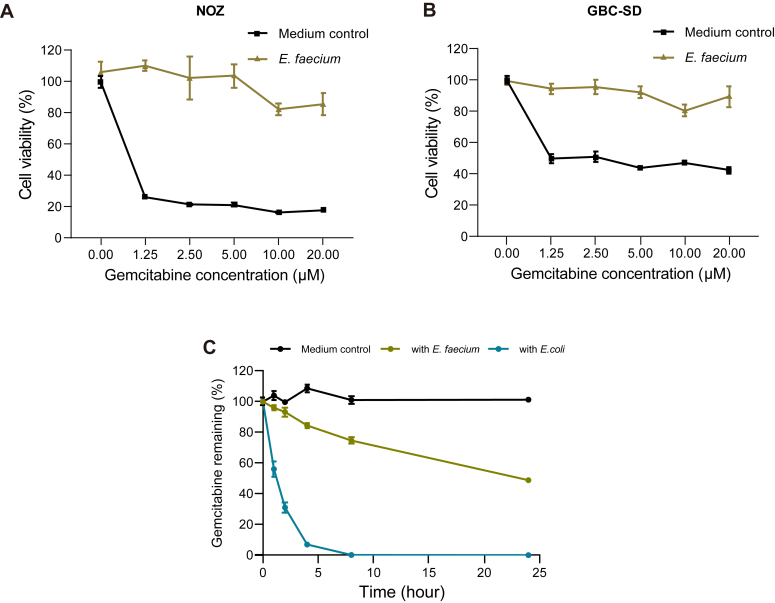
***Enterococcus faecium* drives gemcitabine resistance in GBC cells.***A* and *B*, viability of NOZ cells (*A*) and GBC cells (*B*) was assessed using the CCK-8 assay after treatment with *E. faecium* and gemcitabine. Various concentrations of gemcitabine were incubated with bacteria (or a bacteria-free medium as a control) in DMEM at 37 °C for 4 h. After removing the bacteria, the residual medium was applied to GBC cells, and cell viability was evaluated using the CCK-8 kit after 72 h. *C*, gemcitabine levels in the MRS broth were monitored using HPLC-MS/MS. A solution containing 4 μM gemcitabine and 10^∧^7 bacteria in MRS Broth was prepared. At various time intervals, bacteria were removed, and the residual gemcitabine levels were measured. Error bars represent the SD based on three biological replicates, each with two technical repeats. GBC, gallbladder cancer.

Considering the separate locations of EfCDA in *E. faecium* and gemcitabine in the cell culture medium *in vivo*, which could potentially limit direct interaction between EfCDA and gemcitabine, a detailed examination of the role of *E. faecium* in the resistance of GBC cells to gemcitabine was carried out. Therefore, gemcitabine was introduced to a culture medium with or without *E. faecium*, followed by an interaction period of 4 h. The medium was then filtered to remove the remaining bacteria, yielding a clean medium for applying to GBC cells. Subsequent observation of GBC cell behavior over 72 h revealed that the NOZ and GBC-SD cells displayed considerable resistance to gemcitabine when exposed to the medium cultured with *E. faecium*. Cell viability remained at approximately 80% despite increasing gemcitabine concentration from 1.25 μM to 20 μM (Fig. 2, *A* and *B*). Contrarily, the control group, grown in a medium devoid of *E. faecium*, displayed a significant drop in cell viability.

To further investigate whether *E. faecium* metabolized gemcitabine, we conducted an experiment where the bacteria were incubated with the drug in MRS broth. *Escherichia coli* expressing D-CDA served as a positive control to compare their degradation effects on gemcitabine. Bacterial samples were filtered at specified intervals, and HPLC-MS/MS was used to quantify the remaining gemcitabine. The results indicated a significant reduction in gemcitabine levels over time in the *E. coli* group, consistent with previous research (8). In contrast, *E. faecium* showed a slower decrease in gemcitabine levels (Fig. 2*C*). The control group, which had gemcitabine but no bacteria, did not exhibit any significant reduction. These outcomes validate that *E. faecium* has the ability to break down gemcitabine, albeit with less efficiency compared to *E. coli*.

### EfCDA possesses the activity to catalyze gemcitabine to dFdU

**Figure 3:**
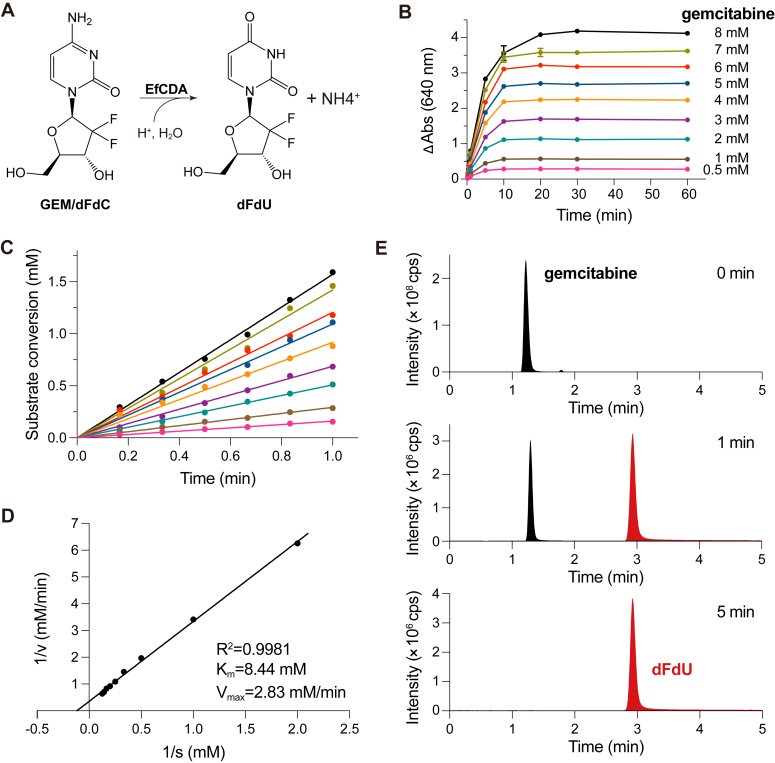
**EfCDA exhibits capability to catalyze gemcitabine to dFdU.***A*, diagram illustrating the catalytic reaction of EfCDA converting cytidine to uridine and producing NH_4_^+^ as a byproduct. *B*, kinetics of the EfCDA enzyme were assessed using the indophenol blue assay. Absorbance at 640 nm was monitored over time with various gemcitabine substrate concentrations in the presence of 0.2 μM EfCDA enzyme. Data were collected from three replicates, and error bars represent the SEM. Absorbance values were normalized by subtracting the background absorbance without the enzyme. *C*, in the initial minute of the reaction, EfCDA-catalyzed gemcitabine kinetics exhibited linear characteristics. *D*, Michaelis–Menten constants for the deamination reaction by EfCDA. Kinetics data were used to create the Lineweaver-Burk plot and for curve fitting using GraphPad Prism software. Error bars represent the SEM. *E*, HPLC-LC/MS chromatography profiles demonstrated the conversion of gemcitabine to its metabolite dFdU during incubation with EfCDA at various time points.

The speed of a reaction is directly influenced by the concentration of enzyme and substrate, with an increase in either enhancing the reaction speed until it reaches saturation. Determining the minimum effective enzyme concentration for gemcitabine degradation by EfCDA is essential in understanding the resistance mechanisms triggered by *E. faecium* in GBC cells. The indophenol colorimetric assay was employed to assess the catalytic activity of EfCDA accurately. The catalytic activity of CDA involves the conversion of cytidine to uridine, generating NH_4_^+^ as a byproduct (Fig. 3*A*). NH_4_^+^ then reacts with phenol, resulting in the formation of indophenol, which exhibits absorption at 640 nm. Previous research has demonstrated that D-CDA, but not T-CDA, can convert gemcitabine to dFdU (8). Hence, we assumed that EfCDA would exhibit similar transformative capacity while producing NH_4_^+^ as a byproduct (Fig. 3*A*).

The kinetic characterization of EfCDA and its substrate, gemcitabine, revealed typical saturation behavior following the Michaelis–Menten model. A concentration of 0.2 μM EfCDA was found to catalyze gemcitabine within the range of 0.5 to 8 mM, achieving an enzyme/substrate concentration ratio of 1:40,000 (Fig. 3*B*). Even at gemcitabine concentrations as high as 7 mM, the reaction attained saturation in approximately 10 min, indicating the robust enzymatic activity of EfCDA. During the initial minute of the reaction, the kinetics of gemcitabine catalyzed by EfCDA exhibited linear characteristics, as evidenced by the obtained *p* values being all less than 0.0001 (Fig. 3*C*). Consequently, the kinetic data obtained within the 0 to 1 min timeframe were utilized to calculate the corresponding Michaelis–Menten constants, resulting in a K_m_ value of 8.44 mM and a V_max_ value of 2.83 mM/min (Fig. 3*D*).

Moreover, the enzymatic activity of EfCDA across varied temperatures and pH conditions was investigated. The enzymatic activity was quantified across a temperature spectrum of 17 to 97 °C at pH 7.4. The peak performance of EfCDA was recorded at 37 °C, with a discernible decrease in catalytic activity beyond this point (Fig. S1A). In addition, the sensitivity of EfCDA to pH variations was assessed across a scope of pH values from 4.0 to 10.0 at a stable temperature of 37 °C. The results indicated that EfCDA exhibited good tolerance to changes in pH (Fig. S1B).

Furthermore, HPLC-MS/MS technology was employed to analyze the catalytic interaction of EfCDA with gemcitabine. An internal standard of [^13^C_1_,^15^N_2_]-gemcitabine and [^13^C_1_,^15^N_2_]-dFdU was introduced in the sample to determine gemcitabine consumption and dFdU production. Initially, a singular peak of gemcitabine was identifiable within the reactive milieu (Fig. 3*E*). However, subsequent examinations showcased a decline in gemcitabine levels, succeeded by an increase in dFdU level over time. By the 5-min mark, a complete conversion of gemcitabine to dFdU was observed, pointing towards EfCDA’s ability to catalyze this transition.

### The overall structure of EfCDA in its apo form

**Figure 4:**
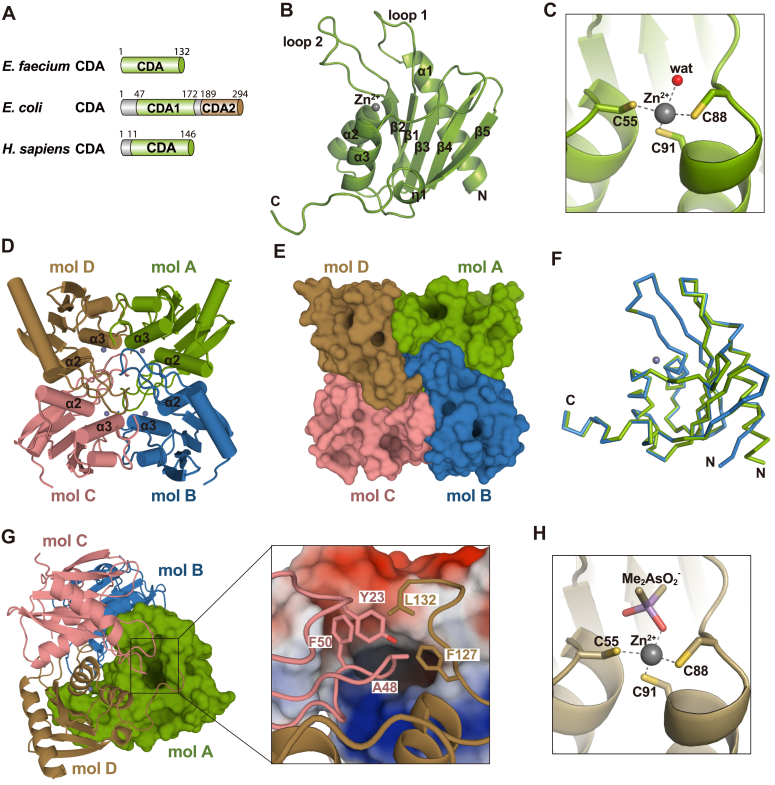
**Structure of the Apo form of EfCDA.***A*, representation of the domain structure of CDA enzymes from *Enterococcus faecium*, *Escherichia coli*, and *Homo sapiens*. *B*, the overall structure of EfCDA is depicted in cartoon mode. *C*, a zinc ion is chelated by C55, C88, C91, and a water molecule in EfCDA's crystal structure. *D*, demonstration of the biological assembly of EfCDA, existing as a homotetramer. *E*, surface representation of the EfCDA homotetramer. *F*, superposition of the EfCDA ribbons from molecules A and B. *G*, a potential gemcitabine-binding pocket is formed by molecules A, C, and D. *H*, chelation of a zinc ion by C55, C88, C91, and a cacodylate ion in EfCDA's crystal structure in apo form II. CDA, cytidine deaminase.

Although structures of bacterial CDAs have been previously elucidated, the structure of *E. faecium* CDA remains unsolved, and no crystal structure of CDA in complex with gemcitabine has been reported. Crystallization trials were carried out to probe the molecular mechanism underlying the EfCDA-catalyzed conversion of gemcitabine to dFdU. The full-length EfCDA protein, encompassing a singular CDA catalytic domain consisting of 132 amino acids, was purified (Figs. 4*A* and S2). Consequently, the crystal structures of EfCDA were determined to a resolution of 1.75 Å.

The structural architecture of EfCDA is composed of a five-stranded β-sheet (β1-β5) encircled by three α-helices (α1-α3) (Fig. 4*B*). Moreover, the protein encompasses two elongated loops that function to connect α1 and β1 (loop 1), as well as β2 and α2 (loop 2), respectively. A zinc-dependent characteristic is exhibited by EfCDA, wherein the crystal structure revealed that a zinc ion was coordinated by three cysteine amino acids (C55, C88, and C91) and a water molecule (Fig. 4*C*).

By previously elucidated crystal structures of CDAs from bacteria and yeast (31, 32), the crystal structure of EfCDA also manifested the formation of a tetrameric assembly (Fig. 4, *D* and *E*). Molecules A and B were in one asymmetric unit with an r.m.s.d. of 0.125 Å (Fig. 4*F*), and the complete tetramer was obtained through a symmetry-related operation. The interface area observed between molecules A and B was 1224.8 Å^2^, while molecules A and D measured 943.2 Å^2^, indicating a stable tetramer. The tetrameric structure of EfCDA is assembled through intermolecular interactions among specific residues in disparate subunits, particularly the α2-α2 and α3-α3 regions of neighboring molecules (Fig. 4*D*). Consequently, the enzyme's active site is encircled by specific residues, including Y23 from loop 1 on molecule C, A48 and F50 from loop 2 of molecule C, and F127 and L132 from the C-terminal loop of molecule D, which may facilitate the regulation of substrate and product ingress and egress (Fig. 4*G*). An additional apo structure of EfCDA was obtained with a high resolution of 1.56 Å, revealing that the zinc ion coordinates with the carbonyl group of cacodylate and the three cysteine residues (Figs. 4*H* and S3). Notably, the distance between the zinc ion and the cacodylate carbonyl group was 2.0 Å, providing insights into the binding mode between EfCDA and its substrate or product.

### Recognition of gemcitabine by EfCDA

The investigation of the interaction between EfCDA and gemcitabine involved the co-crystallization of EfCDA and gemcitabine. Due to the similar molecular structure shared by gemcitabine and dFdU, distinguishing between these two molecules based solely on the electron density map pattern posed a challenge. However, considering the catalytic activity of EfCDA, it was anticipated that gemcitabine would be rapidly converted to dFdU. As a result, the molecule identified in the co-crystallized crystals was determined to be dFdU. Subsequently, the high-resolution crystal structures of EfCDA in complex with dFdU were determined at 1.85 Å, with all associated statistics falling within a reasonable range (Table 1).

**Table 1:** Data collection and refinement statistics

	EfCDD apo-I	EfCDD apo-II	EfCDD-dFdU
Data collection			
Space group	P3_2_21	P2_1_2_1_2	P3_2_21
Cell dimensions			
*a*, *b*, *c* (Å)	83.3, 83.3, 81.7	54.3, 55.7, 82.2	82.9, 82.9, 81.6
α, β, γ (°)	90, 90, 120	90, 90, 90	90, 90, 120
Resolution (Å)	30.0–1.75 (1.78–1.75)^a^	30.0–1.56 (1.59–1.56)^a^	30.0–1.75 (1.78–1.75)^a^
*R*_merge_	0.142 (0.327)^a^	0.124 (0.194)^a^	0.126 (0.401)^a^
*I*/σ*I*	25.5 (8.7)^a^	43.4 (21.7)^a^	48.5 (7.2)^a^
Completeness (%)	89.4 (99.9)^a^	99.1 (97.1)^a^	100.0 (100.0)^a^
Redundancy	17.7 (16.4)^a^	12.4 (11.0)^a^	18.9 (16.0)^a^
Refinement			
Resolution (Å)	29.1–1.75	28.3–1.56	29.1–1.75
No. of reflections	29,869	36,093	33,271
*R*_work_/*R*_free_	0.183/0.209	0.152/0.174	0.161/0.186
*B*-factors	17.9	9.62	24.3
Protein (Å^2^)	16.0	7.01	22.6
dFdU (Å^2^)			21.7
Zn^2+^ (Å^2^)	12.4	4.60	18.0
Water (Å^2^)	30.4	23.0	35.2
R.m.s. deviations			
Bond length (Å)	0.009	0.008	0.007
Bond angles (°)	1.099	1.088	0.979
Ramachandran plot			
Favored (%)	99.6	99.6	100
Allowed (%)	0.4	0.4	0
Outliers (%)	0.0	0.0	0

a Values in parentheses are for the highest-resolution shell.

**Figure 5:**
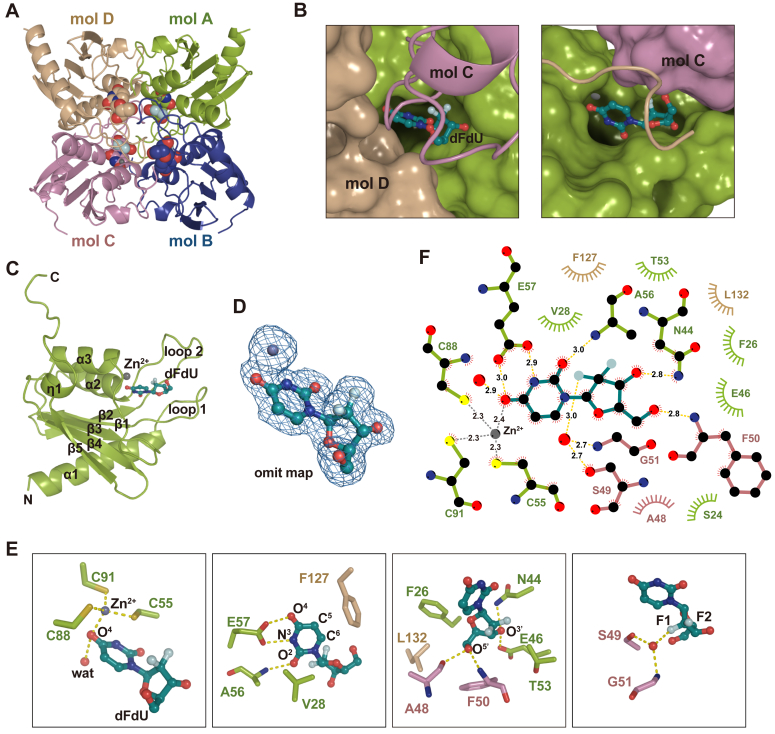
**Crystal structure of EfCDA in complex with dFdU.***A*, overall structure of EfCDA homotetramer in complex with dFdU. *B*, dFdU is inserted into the pocket created by molecules A, C, and D. In the *left* panel, surface representations for molecules A and D are provided. Similarly, on the *right* panel, molecules A and C are shown in surface mode. *C*, cartoon representation of EfCDA complexed with dFdU. *D*, the Polder omit map shows the electron density of dFdU and zinc ion. *E*, detailed interactions between EfCDA and dFdU. *F*, schematic representation of interactions between EfCDA and dFdU.

The dFdU molecule, situated within a pocket delineated by molecules A, C, and D, exhibited interaction interface areas measuring 176.4 Å^2^, 70.8 Å^2^, and 61.0 Å^2^, respectively (Fig. 5, *A–C*), suggesting that these individual molecules all contribute to the binding of dFdU. The omit map for dFdU reveals explicit electron density for dFdU (Fig. 5*D*). Additionally, the O^4^ of the dFdU molecule, working in unison with C55, C88, and C91, demonstrated chelation with Zn^2+^ (Fig. 5, *E* and *F*). Hydrogen bond formation was observed between E57 and O^4^ and N^3^ of dFdU (Fig. 5*E*). O^2^ partakes in a hydrogen bond interaction with the amide bond of A56. The ribose ring of dFdU participates in the creation of multiple hydrogen bond interactions, wherein the O^5^′ forms hydrogen bonds with the amide group of A48 and F50, respectively, and the O^3^′ interacts with the side chains of N44 and E46 via hydrogen bonds. Notably, the two fluorine atoms of dFdU establish hydrogen bonds with the hydroxyl group of S49 and the amide nitrogen of G51 via a water molecule. These extensive hydrogen bonding interactions arguably play a significant role in reinforcing the stable formation of the complex. Furthermore, the dFdU molecules were discovered to interact with F26, V28, T53, F127, and L132 through hydrophobic interactions. A schematic plot generated by LigPlot renders these interactions illustratively (Fig. 5*F*).

### Mutation of key residues fails to induce gemcitabine resistance in GBC cells

**Figure 6:**
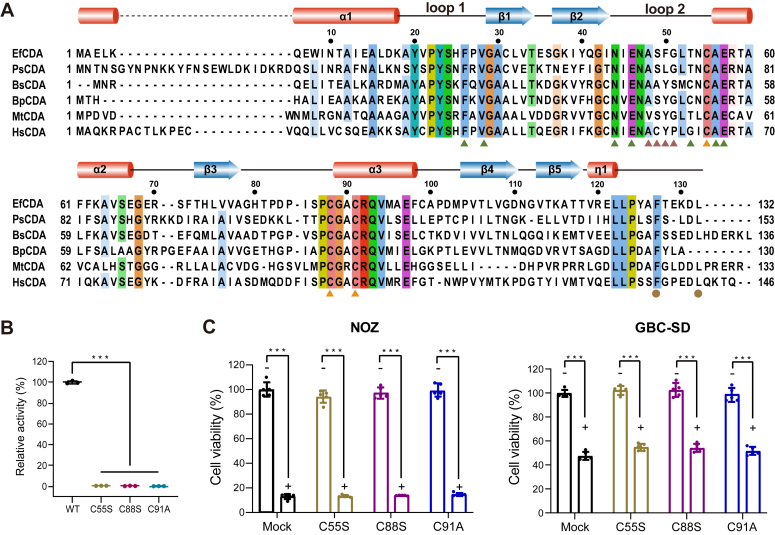
**Validation of key residues in EfCDA’s activity to induce gemcitabine resistance in GBC cells.***A*, structure-based sequence alignment of EfCDA (UniProt code: A0A133N269) with PsCDA (E0E431), BsCDA (P19079), BpCDA (Q63IV7), MtCDA (P9WPH3), and HsCDA (P32320). Elements of secondary structures are marked above the sequence. Key residues engaged in zinc chelating are denoted by *orange triangles*. Residues responsible for the recognition of dFdU are presented with a *green triangle*, *deep salmon triangle*, and *brown circle*. *Ef, Enterococcus faecium* (WP_002291109.1); *Ps, Peptostreptococcus stomatis* (WP_007790209.1); *Bs*, *Bacillus subtilis* (WP_003230049.1); *Bp, Burkholderia pseudomallei* (WP_004187960.1); *Mt, Mycobacterium tuberculosis* (WP_003417264.1); *Hs, Homo sapiens* (NP_001776.1). *B*, after co-culture of gemcitabine (1 mM) and WT or mutant EfCDA (2 μM) for 1 h at 37 °C, the enzyme activity was measured using the indophenol blue assay. *C*, the effects of EfCDA mutants (EfCDA-C55S, C88S, and C91A) and gemcitabine (GEM) on GBC cell viability was assessed using the CCK-8 assay. Measurements were taken after 72 h of exposure to EfCDA mutants, gemcitabine, or their combination. The results were evaluated using a Student’s *t* test (∗*p* < 0.05, ∗∗*p* < 0.01, and ∗∗∗*p* < 0.001). GBC, gallbladder cancer.

Analysis of sequence alignment revealed that despite the typical T-CDA structure, EfCDA exhibited limited conservation in its sequence compared to other T-CDA sequences, except for key residues within the catalytic center (Fig. 6*A*). To validate the significance of these key residues determined for the deamination activity of EfCDA on gemcitabine, three single mutations were made. The key amino acids, C55, C88, and C91, that bind to the Zinc ion were replaced with serine or alanine, respectively (Fig. S2). As expected, the mutant proteins (C55S, C88S, and C91A) failed to convert gemcitabine and produce NH_4_^+^, as detected by indophenol colorimetric assay (Fig. 6*B*). No dFdU was generated after gemcitabine was reacted with mutant proteins for 1 h as detected by HPLC-LC/MS (Fig. S4). Additionally, the impact of the mutant proteins on the viability of GBC cells was examined by introducing them, alongside gemcitabine, to the cell culture medium. Following 72 h, evaluation via the CCK-8 assay revealed that the co-incubation of gemcitabine with the mutated proteins did not trigger any alterations in the sensitivity of the cancer cells towards gemcitabine compared to controls (Fig. 6*C*).

### EfCDA induces gemcitabine resistance in pancreatic cancer cells

Previous research has indicated *in situ* populations of intratumoral bacteria in pancreatic cancer (9, 33). Among them, the homodimer D-CDA, which comprises two tandem catalytic domains, is predominantly found in gammaproteobacteria and can catalyze the conversion of gemcitabine. Conversely, T-CDA exhibits no such catalytic activity. Although our findings have demonstrated that EfCDA, a member of the T-CDA family, can mitigate the efficacy of gemcitabine in gallbladder cancer cells, it is unclear whether EfCDA also engenders gemcitabine resistance in pancreatic cancer cells.

**Figure 7:**
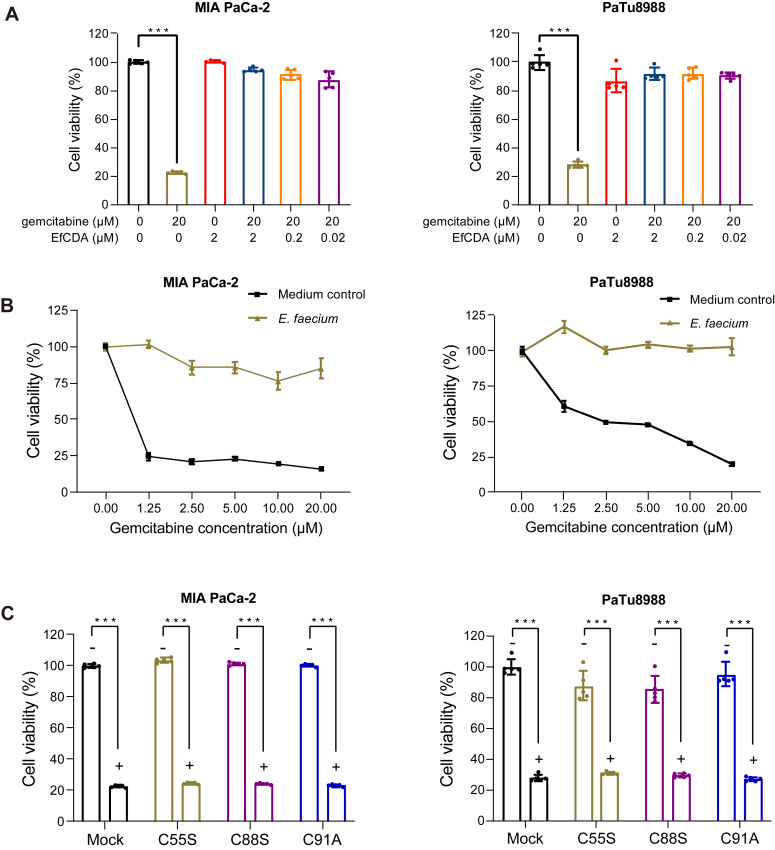
**EfCDA induces gemcitabine resistance in pancreatic cancer cells.***A*, the impact of EfCDA and gemcitabine on the viability of pancreatic cancer cells was investigated. Following 72 h of exposure to various concentrations of EfCDA, gemcitabine (20 μM), or their combination, pancreatic cancer cell viability was assessed using the CCK-8 assay. *B*, pancreatic cancer cells were treated with the filtered medium obtained after incubating various concentrations of gemcitabine with or without bacteria in DMEM at 37 °C for 4 h. After 72 h, cell viability was assessed using the CCK-8 assay kit. Data are presented as means ± SD (n = 3). *C*, after incubating mutant forms of EfCDA with gemcitabine for 2 h, the resulting medium was added to pancreatic cancer cells. Cell viability was assessed using the CCK-8 kit after 72 h.

Pancreatic cancer cell lines MIA PaCa-2 and PaTu8988 were used to examine the effect of EfCDA on gemcitabine resistance. It was found that EfCDA induced gemcitabine resistance in these cells, similar to what was observed in GBC cells (Fig. 7*A*). Co-cultivation of gemcitabine and *E*. *faecium* also reduced the sensitivity of pancreatic cancer cells to the drug, as shown in cell viability experiments (Fig. 7*B*). Additionally, catalytically inactive mutants of EfCDA did not exhibit gemcitabine resistance in cell viability assays, consistent with the findings in GBC cells (Fig. 7*C*). These results suggest that EfCDA functions similarly in pancreatic cancer cells as it does in gallbladder cancer cells, and both T-CDA and D-CDA can metabolize gemcitabine in pancreatic cancer cells.

## Discussion

In our study, we determined the structure of EfCDA, including its monomeric form and complex structures with gemcitabine and the metabolite dFdU. EfCDA exhibits typical characteristics of T-CDA, forming a homotetramer. The primary distinction between T-CDA and D-CDA is that D-CDA possesses two tandem CDA domains (CDA1 and CDA2) and can form a dimer (Fig. 4*A*). Nevertheless, a comparison of its structure with the classic *E. coli* D-CDA (EsCDA) (34), we found a similarity in the catalytic centers of T-CDA and D-CDA, with an r.m.s.d. of 1.22 Å^2^ (Fig. S5, A and B).

Additionally, sequence alignment showed that the key residues involved in catalysis between EfCDA and EsCDA-1 remain conserved. In contrast, EsCDA-2, lacking the key residues that chelate Zn^2+^, serves as a structural scaffold for dimerization (Figs. S5A and S6A). Interestingly, human CDA also exists in two forms. One is similar to T-CDA (hCDA) and can form a homotetramer (35). The other belongs to the AID/APOBEC family, characterized by substrates consisting not of single bases but oligonucleotide chains such as ssDNA or mRNA (26). Some members of this family contain only a single CDA domain, such as AID and APOBEC2 (36), while others possess tandem CDA domains, such as APOBEC3G (37) (Fig. S6B). Although the C-terminal of CDA of AID/APOBEC family proteins has two more α-helices, the overall topology of hCDA and AID/APOBEC CDAs are similar (Figs. S5, and S6, C and D).

Our structural analysis observed that the tetramerization of CDA resulted in the gemcitabine-binding pocket being covered by mol C and mol D (Fig. S3A), thereby limiting the space to accommodate a single nucleotide rather than an entire oligonucleotide chain. Conversely, the CDA of AID is monomeric and contains a positively charged patch near the binding pocket, facilitating the binding of oligonucleotide chains (36) (Fig. S6E). Specific gene editing tools have already integrated Crispr/Cas9 with AID for gain-of-function screening (38). Leveraging these structural insights, it is plausible that the modification of bacterial CDA could be tailored to enable the binding of oligonucleotide chains, thereby holding the potential to enhance and refine current gene editing tools.

In this study, we have also observed that the gut microbiota *E. faecium* and its cytidine deaminase can induce resistance to gemcitabine in two GBC cell lines. Similar effects have also been noted in two pancreatic cancer cell lines. Our findings indicate that T-CDA can metabolize gemcitabine like the previously documented D-CDA in PDAC. The findings suggest that co-administration of antibiotics or deaminase inhibitors with gemcitabine could enhance the efficacy of chemotherapy and address resistance issues. Studies have shown an increase of 11% in overall survival and a 16% improvement in cancer-specific survival for metastatic PDAC patients receiving gemcitabine in combination with antibiotics during chemotherapy. Conversely, similar benefits were not seen in patients treated with fluorouracil (39). This discrepancy implies that antibiotics may play a role in overcoming gemcitabine resistance caused by bacteria, potentially leading to better outcomes in PDAC treatment.

However, gemcitabine resistance involves an intricate interplay of intrinsic and acquired mechanisms, including alterations in drug transporters, metabolic enzymes, and apoptotic pathways that collectively impact the drug's effectiveness (40). Moreover, the tumor microenvironment, influenced by intratumoral microbiota, plays a vital role in modulating gene expression and drug delivery, potentially leading to chemoresistance (10). The ‘TIMER’ framework (Translocation, Immunomodulation, Metabolism, Enzymes, and Microbiota, and their Regulation) has been recently proposed, suggesting that the interaction of these intricate factors can affect the relationship between gut microbiota and these drugs, resulting in diverse clinical outcomes such as improved drug efficacy, compromised therapeutic effects, or increased toxicity (41).

Given the complexity of resistance mechanisms and the emerging role of microbiota in chemoresistance, our study serves as an initial exploration of this intricate field. Further comprehensive research is essential to uncover detailed molecular mechanisms and develop effective strategies to combat chemotherapy resistance.

## Experimental procedures

### Cell culture

The GBC-SD and pancreatic cancer cell lines MIA PaCa-2 and PaTu8988 were obtained from the Cell Bank of the Chinese Academy of Sciences. Another GBC cell line, NOZ, was purchased from Procell Life Science & Technology Co, Ltd. Before use, all cell lines were subjected to STR profiling to confirm the absence of *mycoplasma* contamination. High-glucose Dulbecco’s modified Eagle’s medium (Gibco) was used to culture GBC-SD, MIA PaCa-2, and PaTu8988 cell lines, while William's E (Gibco) was used for NOZ cell line culture. A humidified incubator with 5% CO_2_ at 37 °C was employed for cell line incubation. All cell lines were supplemented with 10% fetal bovine serum (Gibco) and penicillin-streptomycin (HyClone) and maintained in a humidified chamber with 5% CO_2_ at 37 °C.

### Chemical reagents

Gemcitabine (dFdC), gemcitabine-^13^C_1_,^15^N_2_ hydrochloride, 2′,2′-difluorodeoxyuridine, and 2′,2′-difluorodeoxyuridine -^15^C_1_,^15^N_2_ were purchased from Toronto Research Chemicals, and sodium nitroprusside was purchased from Shanghai Yuanye Bio-Technology Co, Ltd. Sodium hypochlorite pentahydrate was obtained from Perfemiker.

### Cloning

The pET28-SMT3a vector containing an N-terminal Ulp1-cleavable 6×His-SUMO tag was utilized for cloning the DNA fragment encoding the cytidine deaminase of *E. faecium* (EfCDA). The EfCDA mutants were introduced utilizing a two-step PCR approach and subsequently integrated into the pET28-SMT3a plasmid. Sequence analyses were performed to validate the correctness of all the created plasmids.

### Protein expression and purification

The cytidine deaminase of *E. faecium*, tagged with an N-terminal Ulp1-cleavable 6×His-SUMO tag, was expressed in *E. coli* BL21 (DE3) cells at 37 °C. The cells were cultivated in LB medium supplemented with 50 mg/ml kanamycin until they reached an optical density of 0.6 at 37 °C. Subsequently, IPTG was added to a final concentration of 0.2 mM, and the cells were further cultured at 18 °C overnight. After harvesting the cells by centrifugation at 4000 rpm for 15 min, the cells were lysed using a French press (JNBIO) at 4 °C.

The 6×His-SUMO–tagged proteins were purified by affinity chromatography using a HisTrap HP column (Cytiva). The Ulp1 protease was used to cleave the 6×His-SUMO tag, and the proteins were then purified by ion exchange chromatography on a HiTrap Q column (Cytiva) and eluted in an NaCl gradient. Finally, the proteins were purified by size-exclusive chromatography on a Superdex G75 HiLoad 16/60 column (Cytiva) in a buffer containing 10 mM Tris, pH 8.0, 100 mM NaCl, and 1 mM DTT.

### Crystallization and structure determination

The protein EfCDA was purified to a concentration of 15 mg/ml, followed by crystallization using the vapor-diffusion method. The reservoir solution employed for growing apo-I crystals comprised 0.1 M Tris at pH 8.5 and 2.2 M (NH_4_)_2_SO_4_, while the apo-II crystals were grown in a reservoir solution consisting of 0.1 M sodium cacodylate at pH 6.5 and 20% PEG 4000.

The crystals of EfCDA complexed with gemcitabine were obtained by mixing the protein with gemcitabine at a 1:10 ratio and then grown under the same conditions as the apo form. The crystals were rapidly frozen in liquid nitrogen with a cryoprotectant buffer comprising the reservoir solution and 15% (w/v) glycerol. Diffraction data were collected at the BL18U1 beamline of the Shanghai Synchrotron Radiation Facility (SSRF) and processed using the HKL3000 software suite (42). The molecular replacement method was employed to solve the structure using the structure generated from AlphaFold (43, 44) as the search model. Model building and refinement were carried out interactively using COOT (45) and Phenix (46). The data collection and refinement statistics are presented in Table 1. The buried surface area was computed using PISA. The PyMOL molecular graphics system, version 1.8 (Schrödinger, LLC), was utilized to present graphic structures (47).

### Cell viability assay

Cancer cells were inoculated into 96-well plates at a density of 2 × 10^3^ cells/100 μl during the logarithmic growth phase for each experimental group. After 72 h of treatment with chemical reagents, 10 μl of Cell Counting Kit-8 solution (Yisen Biology) was added directly to the cells and incubated at 37 °C for 2 h. The absorbance at 450 nm was measured using a spectrophotometer (Thermo Multiskan Sky). The average OD value of three wells was used to calculate the cell survival rate, and the cell viability rate was expressed as a percentage and calculated using the formula [(OD_Treatment_ − OD_Blank_)/(OD_control_ − OD_Blank_)] × 100%.

### Gemcitabine resistance assay in *E. faecium*

The bacterial strain (ATCC19434) was obtained from the BeNa Culture Collection in China and grown overnight in MRS broth from the same collection. The following day, the bacterial culture was diluted 1:25 with Dulbecco’s modified Eagle’s medium and plated onto 96-well U-plates (BD Falcon #353077). Subsequently, different concentrations of gemcitabine were added to the bacteria, and the mixture was incubated at 37 °C for 4 h. After incubation, the bacteria-drug mixture was diluted 1:8 in a 96-well filter plate (Pall #8119) and centrifuged at 1500*g* for 10 min at room temperature in a 96-well absorbent plate (VWR #82051-242). The final filtrate was then added to human gallbladder cancer cells (2 × 10^3^ cells per well), and cell growth was measured using a commercially available cell counting kit-8 (CCK-8; Yisen Biology).

### HPLC-MS/MS detection of gemcitabine and its metabolite

A total concentration of 1 mM gemcitabine was incubated with a purified protein at 0.2 μM in PBS buffer for a specified period at 37 °C. The HPLC-MS/MS method was utilized to determine the percentage of gemcitabine loss compared to pre-incubation levels. Furthermore, at a concentration of 4 μM, gemcitabine was incubated with 1 × 10^7^ bacteria in MRS broth. The bacterial samples were filtered at specified intervals, and the remaining gemcitabine concentration was quantified via HPLC-MS/MS. An internal standard of [^13^C_1_,^15^N_2_]-gemcitabine was added to each sample to a final concentration of 200 ng/ml to determine gemcitabine levels in the samples.

### Analysis of CDA activity

CDA activity was evaluated using the indophenol blue assay, which has been previously described (48, 49). This assay employs two precursor solutions, solution A (0.2 mol/L phenol in 0.4 mM sodium nitroprusside) and solution B (4% sodium hypochlorite in 0.2 mol/L sodium hydroxide solution). This assay aims to determine the extent of the deamination reaction catalyzed by CDA, wherein gemcitabine is converted to 2′,2′-difluorodeoxyuridine with a simultaneous release of ammonium ion in a stoichiometric fashion. The liberated ammonium concentration was measured using spectrophotometry at 640 nm after coupling with phenol.

### Statistical analysis

The statistical analysis of the data was conducted using GraphPad Prism 9 software (https://www.graphpad-prism.cn/). The results are expressed as the mean ± standard error. Differences between groups were evaluated using the two-tailed, unpaired Student's *t* test. Statistical significance was defined as *p*-values less than 0.05. In the figures, the level of significance was indicated using the following notations: ∗*p* < 0.05, ∗∗*p* < 0.01, and ∗∗∗*p* < 0.001.

## Data availability

The structural coordinates have been deposited to the Protein Databank under accession number 8X6W (apo-I), 8X6Y (apo-II), and 8X6U (complex).

## Supporting information

This article contains supporting information.

## Conflict of interest

The authors declare that they have no conflicts of interest with the contents of this article.
