# Supplementary figures and images for: Deciphering the role of Enterococcus faecium cytidine deaminase in gemcitabine resistance of gallbladder cancer
Source: J Biol Chem. 2024 Mar 15;300(4):107171. doi: 10.1016/j.jbc.2024.107171 (PMC11007441; doi:10.1016/j.jbc.2024.107171)

**A**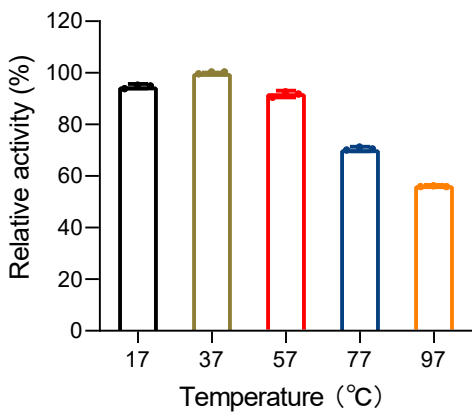**B**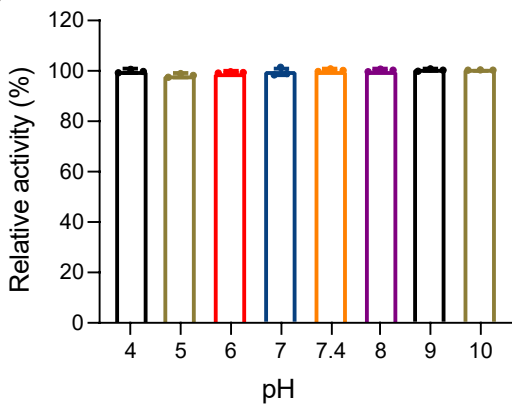

Supplement: Supplemental Figure S1 [file mmc1.pdf]

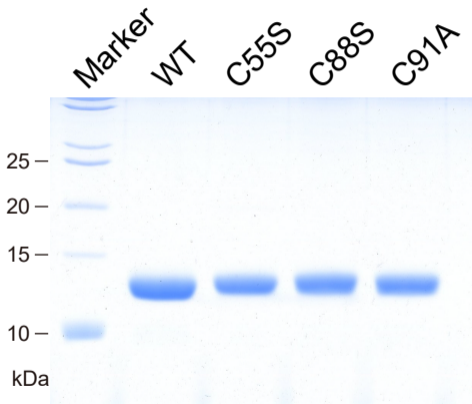

Supplement: Supplemental Figure S2 [file mmc2.pdf]

A

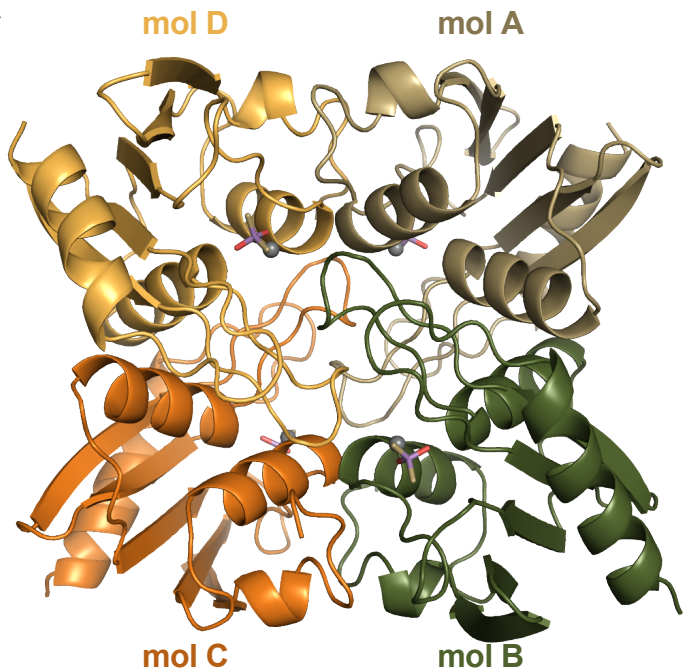

B

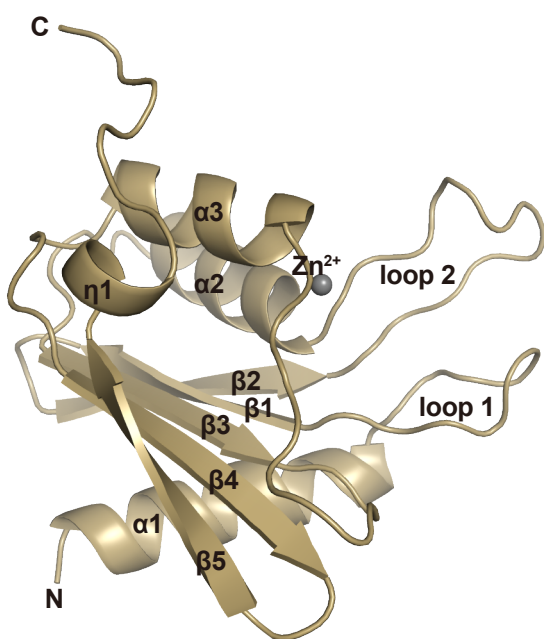

Supplement: Supplemental Figure S3 [file mmc3.pdf]

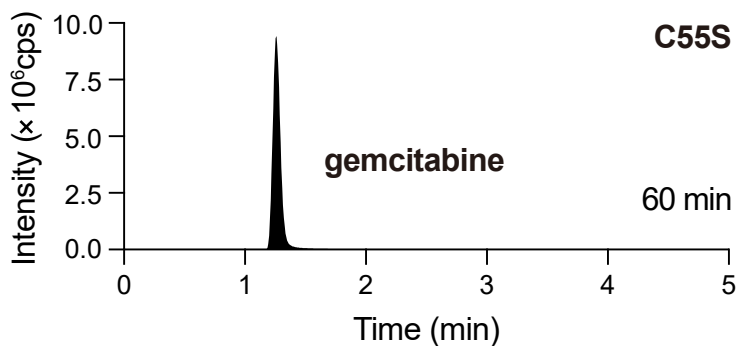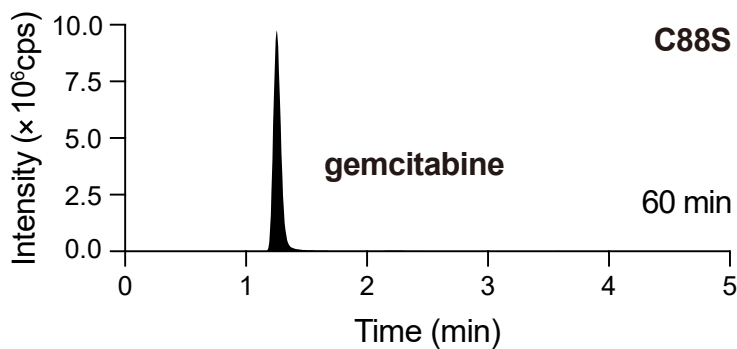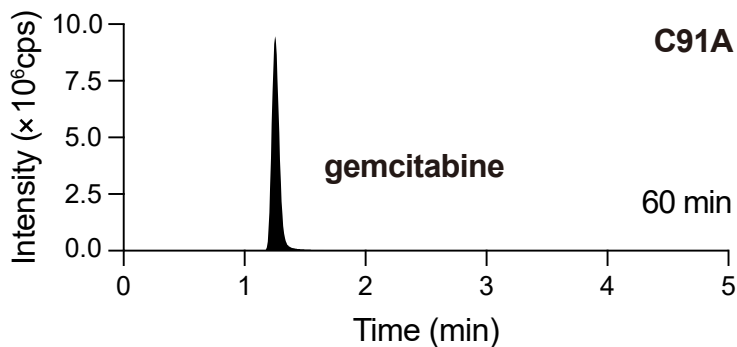

Supplement: Supplemental Figure S4 [file mmc4.pdf]

A

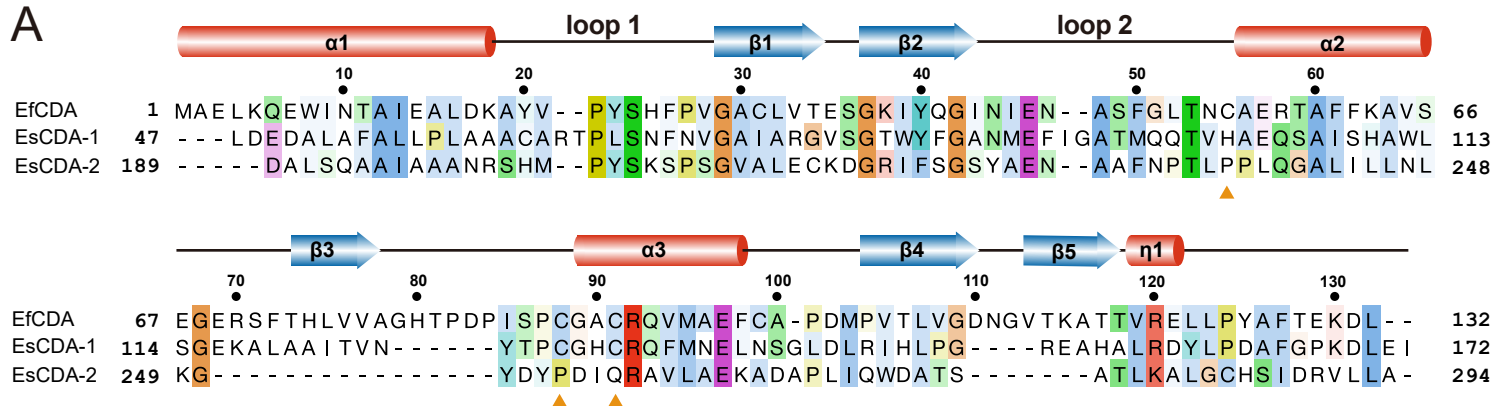

B

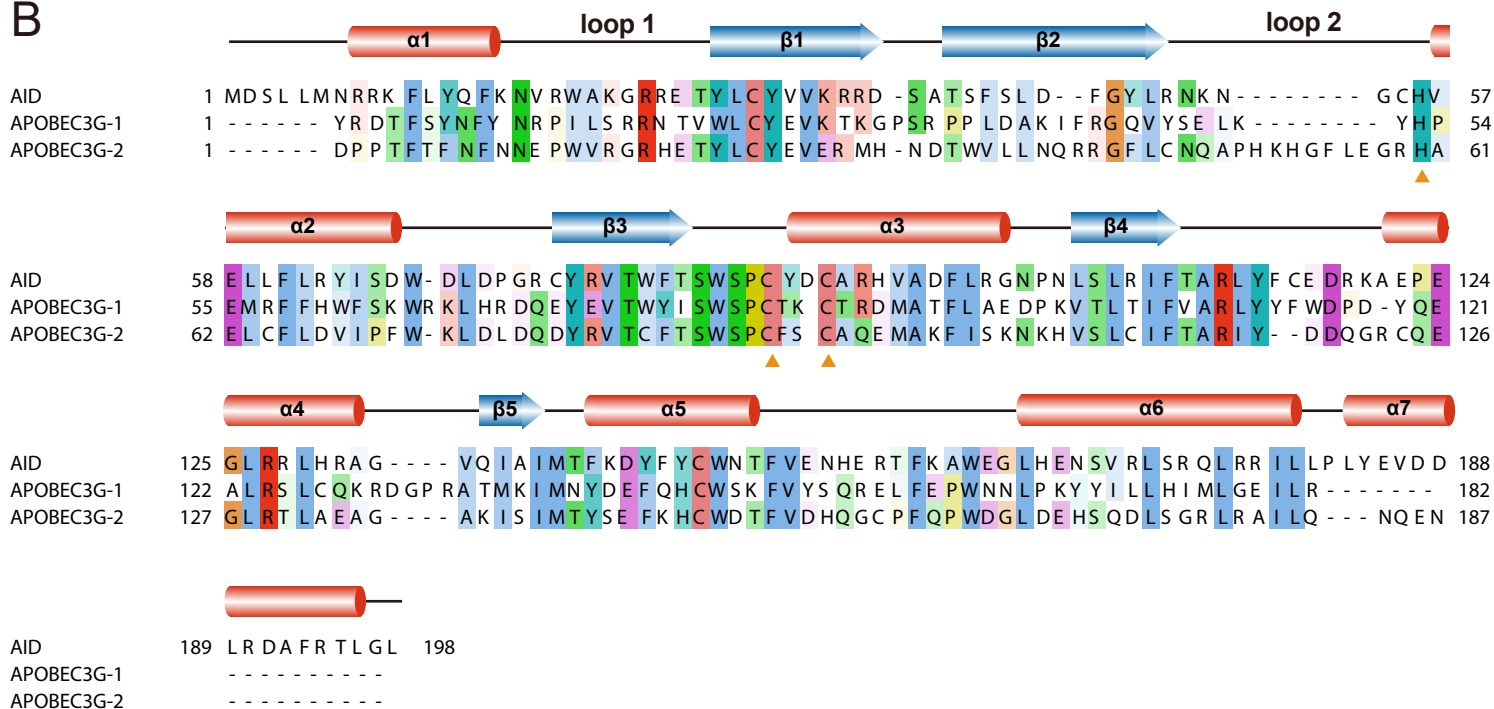

Supplement: Supplemental Figure S5 [file mmc5.pdf]

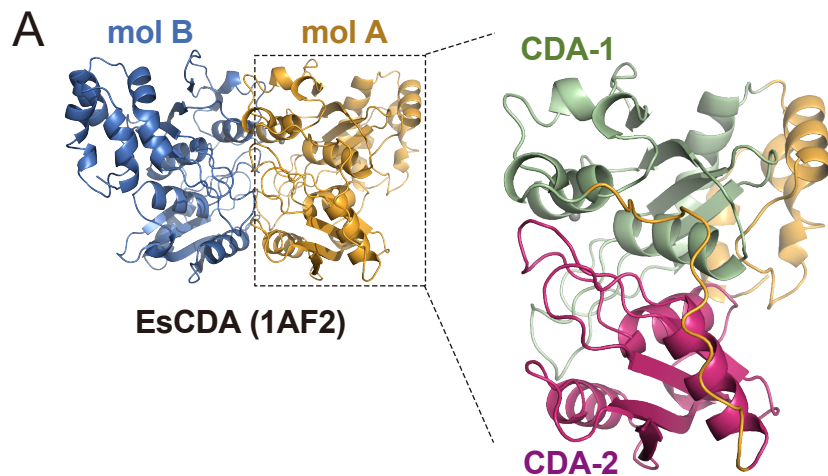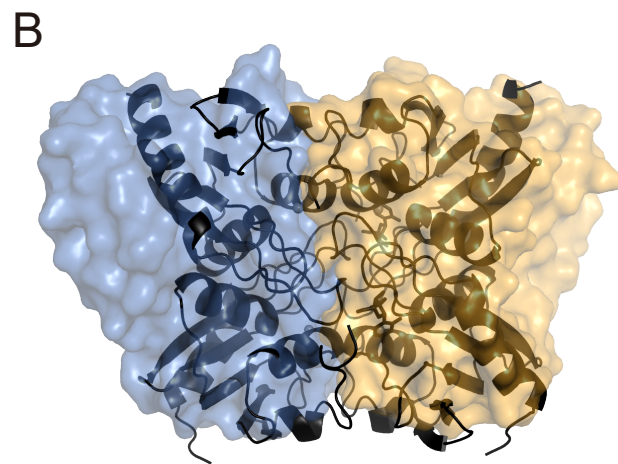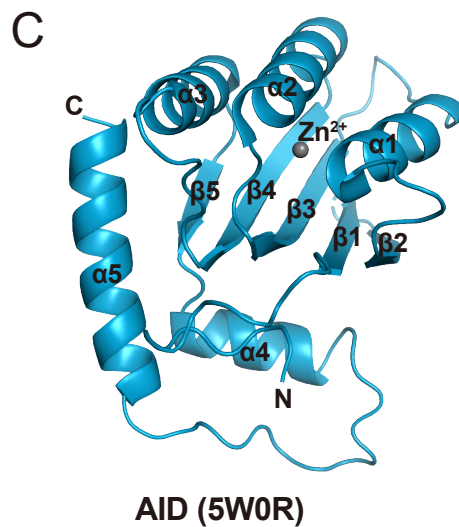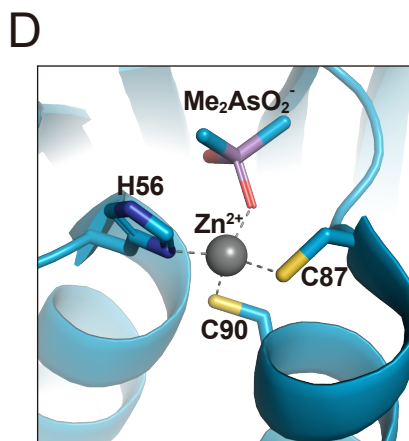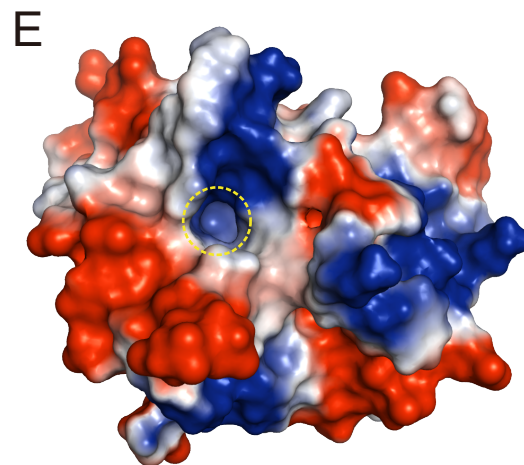

Supplement: Supplemental Figure S6 [file mmc6.pdf]
