# Supplementary material for: Deciphering the role of Enterococcus faecium cytidine deaminase in gemcitabine resistance of gallbladder cancer
Source: J Biol Chem. 2024 Mar 15;300(4):107171. doi: 10.1016/j.jbc.2024.107171 (PMC11007441; doi:10.1016/j.jbc.2024.107171)
Supplement: Supplemental Figure S1–S6 Legends [file mmc7.docx]

**Figure S1. Effects of Temperature and pH on the Enzyme Activity of EfCDA**

(**A**) The influence of temperature on enzyme activity was evaluated. Under optimum pH conditions, the enzyme activity was determined to be between 37 and 97 °C. (**B**) The effect of pH on enzyme activity was assessed using the indophenol blue assay. The enzyme activity was assayed in PBS buffer (pH 4.0–10.0) at 37°C.

**Figure S2. Purity of Wild-type and Mutant EfCDAs.**

The wild-type and mutant EfCDAs were detected by 15% Tricine-SDS-PAGE and visualized by Coomassie Blue staining.

**Figure S3. Structure of EfCDA apo II Form**

(**A**) The homotetrameric biological assembly observed in EfCDA apo form II. (B) The overall structure of EfCDA apo form II is depicted in cartoon mode.

**Figure S4. HPLC-LC/MS Chromatography Profile of Mutant EfCDAs**

A concentration of 20 µM gemcitabine was incubated with purified mutant proteins (C55S, C88S, and C91A) at a concentration of 2 µM in PBS buffer for 1 hour at 37°C. The conversion of gemcitabine to dFdU, catalyzed by the mutant EfCDAs, was monitored using HPLC-LC/MS. No peaks corresponding to dFdU were observed for the C55S, C88S, and C91A mutants.

**Figure S5. Sequence Alignment of Cytidine Deaminase**

(**A**) Sequence alignment of EfCDA (A0A133N269) and the two tandem CDA domains of EsCDA (A0A140N822). Secondary structural elements are indicated above the sequence. (**B**) Sequence alignment of human AID (Q9GZX7) and the two CDA domains of APOBEC3G (Q9HC16). Secondary structural elements are indicated above the sequence.

**Figure S6. Comparative Analysis of Other Cytidine Deaminase Structures**

(**A**) The structure of the homodimeric EsCDA (PDB 1AF2) is presented, displaying the N-terminal (tan), CDA-1 (light green), and CDA-2 (magenta) regions in the zoom-in box. (**B**) Superimposition between the tetrameric EfCDA and the dimeric EsCDA, with EsCDA displayed in surface mode and EfCDA shown in cartoon mode. (**C**) The overall structure of human AID (PDB 5W0R). (**D**) Chelation of a zinc ion by H56, C87, C90, and a cacodylate ion in the crystal structure of AID. (**E**) The electropotential surface of human AID is shown, with the catalytic pocket enclosed by a yellow dashed circle.
